# Constitutive heterochromatin heteromorphism in the Neotropical armored catfish *Hypostomusregani* (Ihering, 1905) (Loricariidae, Hypostominae) from the Paraguay River basin (Mato Grosso do Sul, Brazil)

**DOI:** 10.3897/CompCytogen.v13i1.30134

**Published:** 2019-02-11

**Authors:** Greicy Ellen de Brito Ferreira, Ligia Magrinelli Barbosa, Ana Camila Prizon-Nakajima, Suzana de Paiva, Margarida Maria de Rossi Vieira, Raquel Bozini Gallo, Luciana Andreia Borin-Carvalho, Renata da Rosa, Cláudio Henrique Z wadzki, Isabel Cristina Martins dos Santos, Ana Luiza de Brito Portela-Castro

**Affiliations:** 1 Universidade Estadual de Maringá, Departamento de Biotecnologia, Genética e Biologia Celular, 87020-900 Maringá, Paraná, Brazil; 2 Universidade Estadual do Mato Grosso do Sul, Centro de Ciências Biológicas, 79400-000 Coxim, Mato Grosso do Sul, Brazil; 3 Universidade Estadual de Londrina, Departamento de Biologia Geral, 86057-970, Londrina, Paraná, Brazil; 4 Universidade Estadual de Maringá, Departamento de Biologia/Núcleo de Pesquisas em Limnologia, Ictiologia e Aquicultura (Nupélia), 87020-900 Maringá, Paraná, Brazil

**Keywords:** Chromosome painting, chromosomal polymorphism, chromosome specific probe, FISH

## Abstract

A cytogenetic analysis based on the integration of a number of different chromosomal methodologies, including chromosome microdissection was carried out to characterize the chromosomally polymorphic *Hypostomusregani* population from the Paraguay River basin, state of Mato Grosso do Sul in Brazil. All specimens had 2n=72 (FN=116) but two distinct karyotype formulas: karyomorph A (12m+14sm+18s+28a) and karyomorph B (13m+14sm+17st+28a). Karyomorph A and B differed only for pair 19 that consisted of two subtelocentrics in karyomorph A and a large metacentric and a subtelocentric in karyomorph B. This heteromorphism was due to extensive heterochromatinization of the short arm of the large metacentric, as highlighted by C-banding. The microdissection of the large metacentric of pair 19 allowed the production of a probe, named HrV (*Hypostomusregani* Variant), that hybridized to the whole *p* arm of the large metacentric and the pericentromeric region of the short arm of its (subtelocentric) homologue (karyomorph B) and of both homologs of pair 19 in karyomorph A. Additional cytogenetic techniques (FISH with 18S and 5S rDNA probes, CMA_3_ and DAPI staining) allowed a finer distinction of the two karyomorphs. These results reinforced the hypothesis that the novel large metacentric of *H.regani* (karyomorph B) was the result of the amplification of heterochromatin segments, which contributed to karyotypic diversification in this species.

## Introduction

*Hypostomus* Lacépède, 1803 is the most species-rich catfish genus in the Neotropical subfamily Hypostominae (Loricariidae), which comprises around 135 species (Zawadzki et al. 2016). The species-level taxonomy of this genus is complex, being hampered by the considerable morphological variation found in local populations and the presence of numerous cryptic species with major intraspecific variation in morphology and body pigmentation patterns (Dias and Zawadzki 2018).

The genus *Hypostomus* is cytogenetically highly diversified, with a wide range of diploid (2n=64–84) and fundamental (FN = 82–121) numbers as well as, inter- and intra-specific differences in the number and position of 18S and 5S rDNA clusters (Bueno et al. 2014, Lorscheider et al. 2015, Rubert et al. 2016). In fish, the amount and position of the heterochromatic blocks have been related to the occurrence of chromosomal rearrangements or amplifications, especially during the origin and evolution of specific chromosomes, such as sex chromosomes and B chromosomes (Vicari et al. 2010). However, although scarce, available data on the heterochromatin of *Hypostomus* species indicate a great diversity in its amount and constitution (Artoni and Bertollo 1999, Kavalco et al. 2004, Bittencourt et al. 2011a, Traldi et al. 2012, Baumgärtner et al. 2014, Kamei et al. 2017).

One of the first analyses of the genomic distribution of heterochromatin in *Hypostomus* revealed two general distribution patterns: (i) species with a small amount of heterochromatin, located in subterminal and/or centromeric regions, and (ii) species with a large number of heterochromatic regions located in interstitial sites in several acrocentric chromosomes (Artoni and Bertollo 1999, 2001).

Regarding the molecular composition of heterochromatin in *Hypostomus* species, analysis has demonstrated CG- or AT-rich content (Chromomycin A_3_ or Mithramycin A and 4´-6-Diamin-2-Phenylindole–CMA_3_/DAPI) revealing heterogeneity in these regions, which suggests important implications for the karyotype evolution of this genus (see e.g. Artoni and Bertollo 2001, Kavalco et al. 2004, Rubert et al. 2008, 2011, Milhomem et al. 2010, Maurutto et al. 2013) and other groups of fishes, such as *Gymnotus* Linnaeus, 1758 (Scacchetti et al. 2011), *Bryconamericus* Eigenmann, 1907 (da Silva et al. 2014) and *Ancistrus* Kner, 1854 (Prizon et al. 2016). In addition, analyses with restriction enzymes banding (as AluI, BamHI, HaeIII and DdeI), associated with C-banding technique, revealed heterogeneous heterochromatin patterns in four populations of Hypostomuspropeunae (Steindachner, 1878) (Bittencourt et al. 2011a) and the existence of distinct evolutionary units in allopatric populations of Hypostomuspropewulchereri (Günther, 1864) (Bittencourt et al. 2011b).

The ichthyofauna of the Paraguay River is still poorly-studied, although 14 *Hypostomus* species are known to occur in this basin (Cardoso et al. 2016). *Hypostomusregani* (Ihering, 1905) was originally described for specimens collected in the Piracicaba River (Upper Paraná River basin), but it has also been reported for the Upper Paraguay basin (Zawadzki et al. 2014). Therefore, the present study was aimed to investigate the chromosomal characteristics of the *Hypostomusregani* population from the Upper Paraguay basin which had a chromosomal polymorphism. Some specimens of this population possessed a chromosome heteromorphism due to constitutive heterochromatin expansion in the *p* arm of one of the homologues of pair 19. Both classical and molecular cytogenetic (including chromosome painting) techniques were applied to investigate this heteromorphism.

## Material and methods

### Samples and chromosome preparations

Forty-eight *Hypostomusregani* specimens (23 males, 20 females, and 5 specimens of unidentified sex) were collected from Onça Stream (18°32'18"S, 54°33'43"W), a tributary of the Taquari River, which is part of the Paraguay River basin, located in the municipality of Coxim, in Mato Grosso do Sul State, Brazil. Sampling was authorized by SISBIO (the Brazilian Federal Biodiversity Information and Authorization System), under license number 40510-1. Voucher specimens were deposited in Nupélia (Núcleo de Pesquisa em Limnologia, Ictiologia e Aquicultura) ichthyological collection of Maringá State University (NUP 9820).

Mitotic chromosomes were obtained from kidney cells by the “air drying” method described by Bertollo et al. (1978) at UEMS-UCX (Universidade Estadual do Mato Grosso do Sul, Coxim city) Laboratory. Active NORs sites were evidenced by silver nitrate impregnation (Howell and Black 1980) and the constitutive heterochromatin was detected by the C-banding technique (Sumner 1972) with modifications in the coloring, as proposed by Lui et al. (2012). Fluorescence in situ Hybridization (FISH) with 18S and 5S rDNA probes was based on Pinkel et al. (1986) protocol. The 18S rDNA probe was obtained from *Prochilodusargenteus* Spix & Agassiz, 1829 (Hatanaka and Galetti Jr 2004), whereas the 5S rDNA probe was obtained from *Leporinuselongatus* Valenciennes, 1850 (Martins and Galetti Jr 1999). Both probes were labeled by nick translation using commercially available kits and following manufacturers’ instructions. Biotin-14-dATP (Bio Nick Labeling System, Gibco, BRL) was used for labeling 18S probe and digoxigenin-11-dUTP (DIG-Nick Translation Mix, Roche) for labeling 5S probe. The hybridization signals were detected using avidin-FITC (fluorescein isothiocyanate) for the 18S rDNA probe and anti-digoxigenin-rhodamine for the 5S rRNA probe. The chromosomes were counterstained with DAPI AntiFade solution (ProLong Gold Antifade Mountant with DAPI, Thermo Fisher).

Metaphases were photographed with an epifluorescence microscope (Axioskop, Zeiss) equipped with a digital camera. The chromosomes were identified based on the modified arm ratio (AR) criteria of Levan et al. (1964), and classified as metacentric (m), submetacentric (sm), subtelocentric (st), and acrocentric (a). The fundamental number (FN) was established considering the metacentric, submetacentric and subtelocentric chromosomes as having two arms, and the acrocentric chromosomes, only one.

### Microdissection and amplification

Five heteromorphic chromosomes (the large metacentric of karyomorph B) found in *H.regani* cells were microdissected using an inverted microscope (Olympus IX71) equipped with a mechanical micromanipulator (TH4-100). The microneedles (approximate diameter 0.7 mm) were prepared from glass capillaries using a micropipette puller (Narishige PC-10). The microdissected chromosomes were transferred to 0.5mL microtube and amplified with GenomePlex Single Cell Whole Genomic Amplification WGA4 kit (Sigma). The products of this amplification were reamplified with GenomePlex WGA3 kit (Sigma). In this reamplification reaction with WGA3 kit, the nucleotide digoxigenin 11-dUTP was incorporated with the ratio 7dTTP: 3digoxigenin-11-dUTP to label the chromosome probe. Both procedures with kits (WGA4 and WGA3) were performed according to manufacturers’ instructions. The final products of these reactions was named HrV (*Hypostomusregani* Variant) and used as a probe for FISH experiments on both karyomorphs (A and B), following the protocol of Pinkel et al. (1986).

## Results

**Figure 1.**
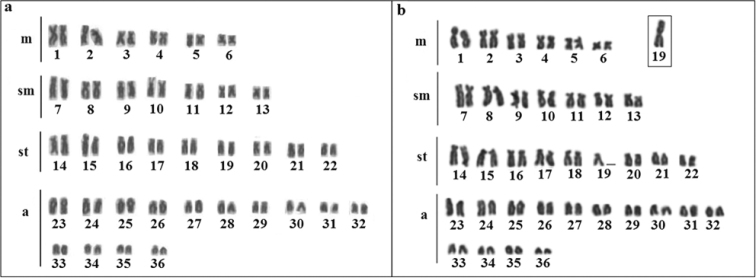
Giemsa stained karyotypes of *Hypostomusregani*: **a** karyomorph A **b** karyomorph B. Scale bar: 10µm.

**Figure 2.**
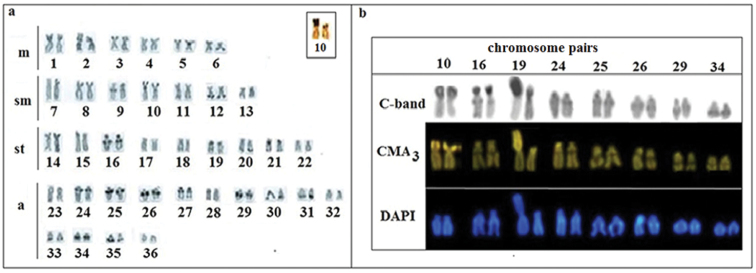
Karyotype of *Hypostomusregani* (karyomorph A) after: **a** C-banding and the NOR-bearing chromosome pair (in box) **b** Some pairs of chromosomes of the karyomorph B showing corresponding bands of C-banding, CMA_3_ and DAPI stained. Scale bar: 10µm.

All *Hypostomusregani* specimens had a diploid number of 72 chromosomes (FN=116), but two different karyotypic formulas. The majority (27) of the specimens had a karyotypic formula of 12m+14sm+18st+28a, named karyomorph A, whereas the remaining 21 specimens had a formula of 13m+14sm+17st+28a, named karyomorph B (Figure 1a, b). Karyomorph B was characterized by a chromosome heteromorphism due to the presence of a large metacentric chromosome (the largest of the complement) and a subtelocentric chromosome corresponding to pair 19 (Figure 1b). This heteromorphism was observed in both males and females, and it was present in 43.74% of the analyzed specimens. Regarding pair 19, C-banding revealed that to the whole *p* arm of the large metacentric of karyomorph B was entirely heterochromatic (Figure 2b), whereas its subtelocentric homologue had heterochromatin in pericentromeric position. This latter pattern also characterized subtelocentric of pair 19 in karyomorph A (Figure 2a). Constitutive heterochromatin was also identified in interstitial positions in pairs 2, 9, 16, 24, 25, 26, 27, 29 and 34 and in subterminal positions in the other chromosomes of both karyomorphs (Figure 2a). C-banding also revealed extensive CMA_3_-positive blocks in chromosomal pairs 10 and 19 (Figure 2b). In contrast, the interstitial heterochromatic blocks in pairs 16, 24, 25, 26, 29 and 34 were negative for CMA_3_ and positive in DAPI (Figure 2b).

**Figure 3.**
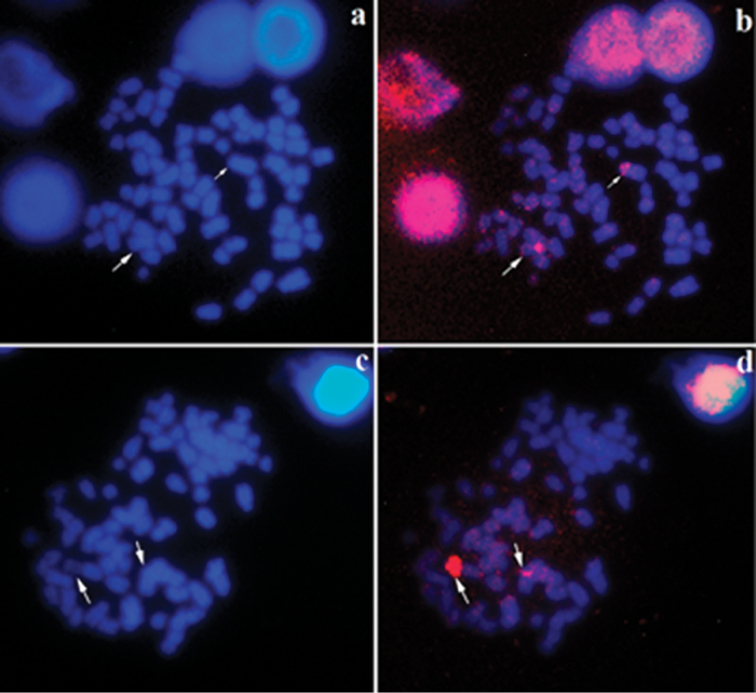
Metaphases of *Hypostomusregani* after FISH with the HrV probe: karyomorph A (**a, b**) and karyomorph B (**c, d**) **a** DAPI stained metaphases of karyomorph A and the arrows indicate pair 19 **b** merged image of metaphase showing intense fluorescent signals positive for HrV probe in the pair 19 (arrows) **c** DAPI stained metaphases of karyomorph B and the arrows indicate heteromorphic pair 19 **d** merged image of metaphase showing intense fluorescent signals positive for HrV probe in the heteromorphic pair 19 (arrows). Scale bar: 10µm.

FISH experiments with HrV probe derived from the heteromorphic metacentric chromosome of karyomorph B revealed two equal-sized signals on the short arm of the two subtelocentric pair 19 of karyomorph A (Figure 3b), coinciding with heterochromatic blocks (Figure 2a). For karyomorph B, the HrV probe revealed a larger fluorescent signal throughout the short arm of the heteromorphic metacentric and on the short arm of the subtelocentric chromosome, the homologous of the pair 19 (Figure 3d). To better visualize the morphology of the pair involved in the heteromorphism, we also showed these metaphases stained in DAPI (Figure 3a, c). Other hybridization fluorescent signals of this probe were observed in several chromosomes of the complement, but they were small and scattered, and did not represent a consistent pattern for the analyzed metaphases (Figure 3b, d).

**Figure 4.**
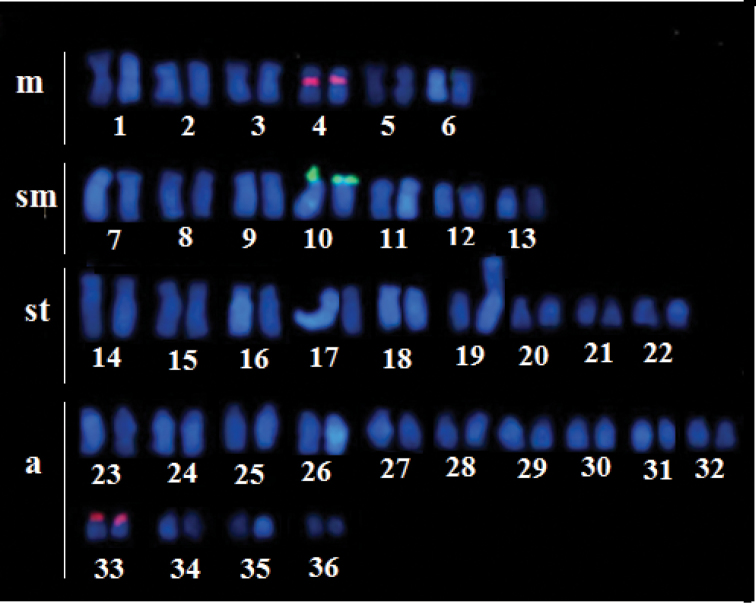
Karyotype of karyomorph B of *Hypostomusregani* after FISH with rDNA probes. Green: 18SrDNA probe; red: 5S rDNA probe. Scale bar: 10µm.

NORs were located in subterminal position on the short arm of submetacentric pair 10, as revealed by the Ag-NOR (Figure 1a, box) and 18S rDNA-FISH (Figure 4) techniques. The 5S rRNA sites were observed in the pericentromeric region of pairs 4 and 33 (Figure 4). These ribosomal sites were observed in both karyomorphs.

## Discussion

The cytogenetic comparison analysis of the *H.regani* population from the Paraguay River with other previously studied populations showed a constant diploid number (2n = 72) for this species. Despite the uniform diploid number in this species, this comparison highlighted different karyotype formulas, including those of the present study, as well as differences in the position and amount of rDNA clusters (18S and 5S). This variability, summarized for *Hypostomus* by Rubert et al. (2016), suggests a cryptic diversity in *H.regani* and the need for a taxonomic revision of this species.

*Hypostomusregani* karyotypes were characterized by a chromosomal polymorphism involving a structural change in a single chromosome pair, subtelocentric pair 19, which resulted in an asymmetry in the karyotype formulas in the analyzed specimens. A similar polymorphism was found in *Hypostomusstrigaticeps* Regan, 1908 (identified as *Hypostomus* sp. B, but subsequently revised by Lorscheider et al. 2015) by Artoni and Bertollo (1999), who reported two distinct karyotypic formulas, 12m+18sm+42st/a and 13m+18sm+41st/a. The formula of the second karyotype differed from the other by the presence of a large metacentric, the largest of the complement, and a median-sized acrocentric chromosome, which corresponded to pair 21 in the homomorphic condition. Their results obtained by C-banding and mithramycin-staining in metaphases containing the large metacentric indicated heterochromatin amplification in one of the ST/A chromosomes.

In the present study, the extensive heterochromatic blocks in the *p* arm of the heteromorphic metacentric chromosome of *H.regani* (karyomorph B) indicate the amplification of repetitive sequences in this region. The fact of the HrV probe has hybridized to the whole *p* arm of the large metacentric, to the pericentromeric regions of the short arm of its (subtelocentric) homologue of karyomorph B and to both homologs of pair 19 in karyomorph A, reinforces the hypothesis that the novel large metacentric of *H.regani* (karyomorph B) was a result of the amplification of heterochromatin segments. The presence of extensive heterochromatic blocks on only one chromosomal arm is an intriguing trait of the chromosome morphology found in some *Hypostomus* species. Heterochromatinization processes and/or an amplification of this region were suggested as an attempt to explain the heterochromatic chromosomal polymorphism in a population of *Hypostomusiheringii* Regan, 1908 (Traldi et al. 2012), in *H.strigaticeps* (Baumgärtner et al. 2014) and Hypostomuspropeplecostomus Linnaeus, 1758 (Oliveira et al. 2015). Furthermore, the presence of transposable elements (TEs) has been confirmed in the heterochromatic regions of a number of fish species (Ferreira et al. 2011), including two *Hypostomus* species (Pansonato-Alves et al. 2013), which could explain the origin of the heteromorphic metacentric in *H.regani*. Finally, heterochromatic chromosomal heteromorphism has been a recurring process in *Hypostomus*, highlighting the role of the heterochromatin in the differentiation of karyotypes, and the potential contribution to chromosome evolution in this group.

The heterochromatic blocks in both karyomorphs of *H.regani* presented heterogeneous composition. Subterminal blocks tended to be rich in GC (CMA_3_^+^, pairs 10 and 19), whereas the interstitial blocks are rich in AT (DAPI+ pairs 16, 24, 25, 26, 29 and 34). It is also interesting to point out that while CMA_3_ blocks are scarce in most *H.regani* chromosomes, the accumulation of GC sequences (CMA_3_^+^) was observed on the short arm of the heteromorphic metacentric of karyomorph B. The homology of the GC-rich sequences on the short arm of the subtelocentric pair 19 of karyomorph A, which presumably represents the original form of the heteromorphic pair of karyomorph B, it further reinforces the hypothesis that the novel large metacentric of *H.regani* (karyomorph B) was the result of the amplification of pre-existing heterochromatin segments.

In a panmictic population, the expected frequency of the chromosomal polymorphism in *H.regani* can be estimated based on the observed frequency of the ST (subtelocentric) and M (metacentric) chromosomes, which were *p* (ST) = 0.78 and *q* (M) = 0.22, respectively. Given a sample of 48 specimens, the expected number of each genotype would be 29.28 ST/ST, 16.32 ST/M, and 2.40 M/M, whereas 27 of the specimens were ST/ST, and 21 ST/M. This represents a significant deviation from Hardy Weinberg Equilibrium (X^2^ = 3.92, d.f. = 1, p < 0.05), although the absence of the M/M genotype may be at least partly due to the small sample size. Alternatively, the M/M genotype may suffer negative selection pressure, determining its absence from the *H.regani* population.

Chromosome mapping data with rDNA sequences are available for few *Hypostomus* species. In this genus, the NORs may be single or multiple, but multiple sites is the most frequent arrangement. This is considered to be a derived trait in Locariids (Bueno et al. 2013, 2014). The mapping of the 18S rDNA gene in other *H.regani* populations has demonstrated multiple sites, located in the terminal position, mostly on the short arms of the st/a chromosomes (Rubert et al. 2016), which contrasts with the findings of the present study, given that the specimens of *H.regani* analyzed here presented single NORs, with active NOR and the 18S rDNA sites located in a terminal position on the short arms of submetacentric pair 10. Thus these chromosomes can be considered markers for the *H.regani* populations from Paraguay River basin.

Chromosomal mapping of 5S rDNA clusters has been carried out for only a few *Hypostomus* species and two patterns have been observed: (i) single 5S-bearing pair has been reported in *Hypostomusiheringii* (Traldi et al. 2012), *H.nigromaculatus* Schubart, 1964 (Traldi et al. 2013) *H.albopunctatus* Regan, 1908 and *H.topavae* Godoy, 1969 (Bueno et al. 2014) and H.propehermanni Ihering, 1905 (Kamei et al. 2017); (ii) multiple sites have been observed in *H.ancistroides* Ihering, 1911 (Traldi et al. 2013, Kamei et al. 2017), *H.affinis* Steindachner, 1877 (Kavalco et al. 2004), *H.cochliodon* Kner, 1854, *H.commersoni* Valenciennes, 1836, *H.faveolus* Zawadzki, Birindelli & Lima, 2008 (Bueno et al. 2014) and *H.topavae* (Kamei et al. 2017). The presence of a centromeric/pericentromeric 5S rDNA sites on the short arm of a metacentric or submetacentric pairs is a frequent feature observed in the most species of *Hypostomus* (Bueno et al. 2014), also detected in the present study to *H.regani*, in pair 4, as well as in pericentromeric position in acrocentric pair 33. These findings indicate a shared condition among *Hypostomus* species that may be a primitive trait (Traldi et al. 2013). However, the number of chromosomes bearing 5S rRNA sites varies among *H.regani* populations, ranging from one pair in the population from Piquiri River (Bueno et al. 2014) to nine chromosomes in the one from Piumhi River (Mendes-Neto et al. 2011). The evolutionary dynamics of the ribosomal genes seems to be related to their association with transposable elements, as observed in some fish species, which indicates that these elements may play a role in the dispersion of the 5S rDNA sites (da Silva et al. 2011, Pansonato-Alves et al. 2013, Gouveia et al. 2017).

## Conclusion

The chromosomal heteromorphism detected in *H.regani* from Onça Stream, in the Taquari River basin, and investigated by chromosome painting provides an important model for the cytogenetic analysis for other species of the genus, in addition to other fish genera in which the role of the heterochromatin in differentiation and evolution of karyotypes need to be better understood. The divergence in karyotype formulas found among different populations of *H.regani* (Rubert et al. 2016) suggests the existence of cryptic species within this taxon, and emphasizes the need of a thorough revision of the taxonomy of this group. While the taxonomic complexity of the genus *Hypostomus* is still far from being sorted out, cytogenetic analyses based on high resolution techniques, such as those applied in the present study, should help to reduce the taxonomic uncertainties in this genus.
